# Comparative Effects of Dextrose and Breast Milk on Physiological Parameters and Crying Duration Among Neonates Undergoing Heel Prick: A Quasi-experimental Study

**DOI:** 10.7759/cureus.84432

**Published:** 2025-05-19

**Authors:** Sabitri Acharya, Pity Koul, Kalpana Sharma

**Affiliations:** 1 Child Health Nursing, School of Nursing Sciences and Research, Sharda University, Greater Noida, IND; 2 Adult Health Nursing, School of Nursing, Chitwan Medical College, Bharatpur, NPL

**Keywords:** breastmilk, dextrose, heel prick, neonates, physiological parameters

## Abstract

Background

Heel pricks are a common but painful procedure in neonatal care, often causing significant distress. Effective pain management is not only essential for the neonate's comfort but also a fundamental ethical obligation of healthcare providers, reflecting a commitment to compassionate and neonatal care. This study aims to compare the effects of dextrose and expressed breast milk in relieving procedural pain, as measured by changes in vital parameters and the duration of crying among neonates while undergoing heel prick.

Methods

A quasi-experimental study was conducted in selected hospitals of Lumbini Province, Nepal, from February 15 to July 2024 among 140 neonates admitted to the neonatal intensive care unit. The neonates were allocated to two groups: the intervention group (n=70), which received 2 mL of 25% glucose, and the expressed breast milk group (n=70), which received 2 mL of expressed breast milk, which was administered two minutes before the heel prick procedure. Physiological parameters were assessed using a biophysiological assessment proforma at baseline and following the heel prick procedure. The biophysiological assessment proforma was validated with reliability coefficients of 0.80. Data analysis was done descriptively and inferentially using non-parametric tests.

Results

The mean birth weight of neonates was 3.07 ± 0.41 kg in the dextrose group and 3.00 ± 0.40 kg in the expressed breast milk group. Vital parameters (heart rate, respiratory rate, and oxygen saturation) stabilized significantly faster in the dextrose group (p < 0.05). The Wilcoxon signed-rank test further confirmed significant within-group changes in physiological parameters across different time points, with a greater effect observed in the dextrose group. In addition, crying duration was shorter in the dextrose group compared to the breast milk group during both heel pricks: 73.14 ± 26.47 vs. 112.85 ± 17.99 seconds for the first heel prick and 67.42 ± 18.23 vs. 98.43 ± 17.33 seconds for the second heel prick.

Conclusion

The study demonstrates that 25% dextrose more effectively stabilized the physiological parameters with less crying duration than expressed breast milk during heel prick.

## Introduction

According to the International Association for the Study of Pain (IASP), pain is defined as an unpleasant sensory and emotional experience associated with, or resembling that associated with, actual or potential tissue damage [1]. For many years, neonatal pain was not given due consideration, as it was widely believed that newborns either did not experience pain or perceived it with less intensity [2]. Consequently, neonatal pain was often underestimated and inadequately managed [3,4]. Hospitalized newborns commonly undergo minor invasive procedures in early life, such as immunizations, heel lancing, bilirubin screening, and blood glucose tests [5-8].

Neonates in neonatal intensive care units (NICUs) often undergo multiple painful procedures daily, with insufficient pain relief. A systematic review of 35 studies found an average of 7.38 painful procedures per day, ranging from two to 17 [9]. In China, 120 neonates experienced 16,840 painful procedures, averaging 13 per day, with over 70% categorized as severely painful [10]. In South India, neonates underwent an average of 8.09 painful procedures daily, primarily heel pricks [11]. Research on extremely preterm infants (<28 weeks) reported an average of 11.24 procedures per day, with only 12.2% receiving analgesia [12]. These findings emphasize the need for improved pain management in NICUs worldwide.

Exposure to painful stimuli during the neonatal period can result in lasting neurobiological effects, including alterations in pain sensitivity, stress response mechanisms, and sensory processing. Early experiences of pain may disrupt the hypothalamic-pituitary-adrenal (HPA) axis and increase glucocorticoid receptor expression in the hippocampus, influencing emotional regulation and stress responses later in life [13]. Repeated painful procedures, especially in the absence of analgesia, have been shown to heighten pain responses, leading to significant increases in vital parameters such as heart rate and respiratory rate, which serve as physiological indicators of distress in neonates. Additionally, neonates, unable to verbalize their discomfort, rely on crying as a primary indicator of pain [14]. These findings underscore the vulnerability of neonates to both immediate and long-term effects of unmanaged pain, highlighting the need for effective, evidence-based pain management strategies to alleviate both physiological stress and distress signals, such as crying.

Effective pain management in neonates is crucial, as repeated exposure to untreated procedural pain can lead to adverse short- and long-term outcomes. While expressed breast milk is often used for its natural soothing properties, its low glucose content and inconsistent availability in NICUs can limit its effectiveness. In contrast, 25% dextrose provides rapid and consistent analgesia through sweet taste-induced endogenous opioid release and is readily available, easy to administer, and more effective in reducing acute pain, as shown in several studies [15,16].

Despite the availability of evidence-based guidelines recommending non-pharmacological methods for managing procedural pain in neonates, their consistent application in NICUs remains challenging. High patient loads, time constraints, and limited staffing often lead healthcare providers to prioritize urgent clinical tasks over pain management interventions. Additionally, methods such as breastfeeding or administering expressed breast milk require maternal involvement, which may not always be feasible due to postnatal recovery, restricted visiting hours, or logistical issues. In such contexts, easily accessible and quick-to-administer interventions like 25% dextrose are more commonly used, even though they too may be inconsistently applied [17,18]. In the context of Nepal, there remains a noticeable gap in comparative studies evaluating non-pharmacological pain relief methods. To address this, the present study was undertaken to evaluate the impact of 25% dextrose and expressed breast milk in stabilizing physiological parameters and reducing crying duration in neonates undergoing heel pricks. Furthermore, the findings aim to contribute to evidence-based neonatal care practices and support the implementation of simple, safe, and cost-effective pain management strategies in resource-limited settings.

## Materials and methods

Research design

This study is a quasi-experimental study with a non-equivalent control group pre-test post-test design to compare the effects of 25% dextrose and expressed breast milk on physiological parameters and duration of crying among neonates while undergoing a heel prick procedure.

Ethical considerations

Written approval was obtained from the authority of Sharda School of Nursing Science and Research, Sharda University, Greater Noida. Ethical clearance was obtained from the Ethical Review Committee of Nepal Health Research Council (NHRC) (reference no: 945) in January 2024. Administrative approval was taken from the medical superintendent and matron of Lumbini Provincial Hospital and Siddhartha Children and Women Hospital, Lumbini Province, Nepal. Written informed consent (assent) was obtained from the mothers of the neonates. Data collection was conducted from February 1, 2024, to June 30, 2024. The study was registered on the Clinical Trial Registry of India with reference number CTRI/2024/01/061630 on January 19, 2024.

Participants

The study included 140 term neonates admitted to the NICU of Lumbini Provincial Hospital and Siddhartha Children and Women Hospital. The neonates were selected based on eligibility criteria and allocated in the approximate ratio of 1:1 to two intervention groups (the 25% dextrose group and the expressed breast milk group). Sample subjects were term neonates aged 0-7 days who were admitted to the NICU regardless of any medical conditions, were on oral feeds with their last feed occurring at least 30 minutes prior, and were in the state of wakefulness. Neonates were excluded if they were on mechanical ventilators and receiving sedatives or had congenital anomalies.

Sample size and sampling technique

The sample size for this study was calculated using G*Power statistical software (ver. 3.1.9.4, Heinrich-Heine-Universität Düsseldorf, Düsseldorf, Germany), with a significance level set at 0.05. Based on the calculations, 70 neonates per group were required to attain 80% power with a 20% error margin. Total enumeration sampling was employed, meaning all eligible neonates who met the inclusion criteria were included in the study, ensuring comprehensive participation.

Intervention

All neonates who met the inclusion criteria were recruited for the study after obtaining relevant characteristics from both the mother and the newborn. Baseline vital signs, i.e., heart rate, respiratory rate, and oxygen saturation (SaO₂), were recorded using a pulse oximeter placed on the neonate's wrist. These measurements were taken using the standard NICU monitor (Edan iM8, Edan Instruments Inc., Shenzhen, China) over four minutes (baseline, immediately after prick, after two minutes, and after four minutes) by the primary investigator, ensuring a stable and accurate baseline for each neonate.

Subsequently, each neonate was assigned to receive one of two interventions: 2 mL of 25% dextrose or 2 mL of expressed breast milk. The dextrose was drawn aseptically from a sterile vial, while the breast milk was freshly expressed by the mother just before the procedure. Both interventions were administered using a sterile dropper by an on-duty staff nurse to maintain uniformity and sterility. If any neonate vomited immediately after administration, they were excluded from the study to avoid compromised absorption of the intervention and potential bias in outcomes.

To account for potential diurnal variations in neonatal response and physiological parameters, the interventions were administered twice, once in the morning and once in the evening. For each intervention time point, pretest assessments of vital signs were recorded before administration, followed by post-test assessments conducted using the same standardized procedure and equipment. This pretest-posttest design enabled a rigorous within-subject comparison of vital parameter changes attributable to the interventions across different times of day.

Blinding

The enrollment and allocation of neonates to the respective intervention groups were carried out by the on-duty staff nurse, thereby ensuring that the primary investigator remained blinded to the type of intervention administered. This blinding was an essential component of the study design, implemented to reduce observer bias and maintain objectivity during the assessment phase. Each neonate received either 2 mL of 25% dextrose or 2 mL of expressed breast milk, which was administered orally using a sterile dropper. Following the administration of dextrose or breast milk, the primary investigator performed the heel prick procedure using a sterile lancet needle. To maintain consistency across all participants and ensure accurate documentation, the entire process from the administration of the intervention to four minutes post-heel prick was continuously video-recorded. This video recording served multiple purposes: it ensured precise timing, offered visual evidence for further analysis, and allowed independent verification of the consistency and integrity of the procedure across all participants. During the observation period, the primary investigator was responsible for monitoring and recording vital physiological parameters, including heart rate, respiratory rate, oxygen saturation, and the duration of crying. This rigorous and standardized approach to data collection was designed to enhance the reliability and validity of the study findings by minimizing variability and ensuring methodological transparency.

Outcome measures

The physiological parameters of the neonates were assessed by a biophysiological assessment proforma, which was developed and validated based on literature and researcher discussion. The primary outcome measure was physiological parameters, and the secondary outcome was duration of crying. This physiological parameter includes maximum heart rate, respiratory rate, and minimum oxygen saturation. The duration of crying was assessed following the heel prick; the time from the first cry until the neonate stopped crying continuously was measured. This was documented using video recordings to ensure accuracy and consistency in assessing the crying duration. The interclass correlation consistency (ICC) of the biophysiological assessment proforma was 0.80. It was assessed at baseline, immediately after the heel prick, and two minutes and four minutes after the heel prick procedure.

Data analysis

Data were arranged and input into SPSS for Windows, Version 16.0 (Released 2007; SPSS Inc., Chicago, Illinois, United States) for analysis. Continuous and categorical data were presented as mean (standard deviation), frequency, and percentage, respectively. Mann-Whitney's test was used to compare means between the groups. The Friedman test was used to compare the changes in physiological parameters within the same group of neonates across multiple time points during each heel prick session. In addition, the Wilcoxon signed-rank test was performed to compare two related measurements taken from the same neonates, specifically, the pre-test and post-test values of vital parameters (such as heart rate and oxygen saturation) within each group. The significance level was set at p < 0.05, with a 95% confidence interval (CI).

## Results

Table 1 presents the baseline characteristics of the neonates in the interventional and control groups. No significant differences were found in variables such as the day of life, gender, birth weight, head circumference, length, and birth order (p > 0.05). However, chest circumference (p < 0.01), APGAR scores at zero and five minutes (p < 0.01), and the reason for admission (p = 0.05) showed significant differences between the groups. Most neonates had normal chest circumference, and respiratory problems were the most common reason for admission.

**Table 1:** Frequency and percentage distribution of neonatal characteristics in the intervention and control groups *A p-value < 0.05 is statistically significant. Mean±SD: mean ± standard deviation; F: frequency; P: percentage; χ²: chi-square; df: degree of freedom

Variables	Interventional		Control		χ² (df)	p-value
	F	P	F	P		
Day of life						
First day	32	45.71	30	42.86	2.10 (2)	0.52
2nd to 3rd day	22	31.42	23	32.86		
4th to 7th day	16	22.86	17	24.28		
Gender						
Male	39	55.7	44	62.9	0.74 (1)	0.39
Female	31	44.3	26	37.1		
Birth weight						
2500–3500	60	85.7	57	81.4	0.46 (1)	0.49
3501–4500	10	14.3	13	18.6		
Mean ± SD	3.07 ± 0.41		3.00 ± 0.40			
Head circumference						
Microcephaly	10	14.3	15	21.4	2.14 (2)	0.37
Normal	59	84.3	55	78.6		
Macrocephaly	01	1.4	00	00		
Length						
Less than normal	07	10.0	00	00	7.36 (1)	<0.01* (S)
Normal	63	90.0	70	100		
Short stature	06	8.6	12	17.1	2.29 (1)	0.13
Normal stature	64	91.4	58	82.9		
APGAR (0 minutes)						
Severely Depressed	29	41.4	50	71.4	13.28 (2)	<0.01* (S)
Moderately depressed	34	48.6	15	21.4		
Excellent	07	10.0	05	7.1		
APGAR (5 minutes)						
Severely depressed	00	00	01	1.4	12.62 (2)	<0.01* (S)
Moderately depressed	16	22.9	35	50.0		
Excellent	34	48.6	34	48.6		
Birth order						
First child	41	58.6	44	62.9	3.77 (2)	0.15
Second child	18	25.7	22	31.4		
Third child	11	15.7	04	5.7		
Reason for admission						
Respiratory problems	31	44.3	44	62.9	4.85 (2)	0.05* (S)
Infections	24	34.3	16	22.9		
Hepatobiliary problems	15	21.4	10	14.3		

Table 2 displays the means, standard deviations, and U-values of vital parameters before and after the intervention in both the intervention and control groups. The results revealed significant changes in heart rate, respiratory rate, and oxygen saturation between the groups. Specifically, the intervention group showed a significant increase in heart rate from 127.62 ± 12.97 bpm to 136.53 ± 13.01 bpm (U = 1334.00, p < 0.01), a slight increase in respiratory rate from 52.68 ± 7.87 to 54.52 ± 7.49 (U = 1727.50, p = 0.03), and a significant decrease in oxygen saturation from 99.23 ± 1.20% to 96.65 ± 1.17% (U = 512.50, p < 0.01). In contrast, the control group showed a smaller increase in heart rate from 123.74 ± 11.52 bpm to 147.16 ± 12.47 bpm, with no significant change in respiratory rate and a decrease in oxygen saturation from 98.87 ± 1.74% to 94.40 ± 1.56%, which was statistically significant. These findings indicate that the intervention group exhibited more pronounced changes, particularly in heart rate and oxygen saturation, compared to the control group.

**Table 2:** Comparison of the mean vital parameters between the intervention and control groups A p-value < 0.05 is statistically significant. M±SD: mean ± standard deviation; U-value: Wilcoxon rank-sum test

Variables	Groups	Pre-test			Post-test		
		M±SD	U-value	p-value	M±SD	U-value	p-value
Heart rate	Intervention	127.62±12.97	2048.00	0.93	136.53±13.01	1334.00	<0.01
	Control	123.74±11.52			147.16±12.47		
Respiratory rate	Intervention	52.68±7.87	2067.50	0.10	54.52±7.49	1727.50	0.03
	Control	54.80±6.77			58.46±6.66		
Oxygen saturation	Intervention	99.23±1.20	2306.50	0.49	96.65±1.17	512.50	<0.01
	Control	98.87±1.74			94.40±1.56		

Table 3 compares the mean crying duration (in seconds) between the intervention and control groups across two sessions. In both sessions, neonates who received the intervention of 25% dextrose had a significantly shorter crying duration compared to those who received expressed breast milk. In Session I, the intervention group had a mean crying duration of 73.14 ± 26.47 seconds, while the control group had 112.85 ± 17.99 seconds. Similarly, in Session II, the intervention group cried for 67.42 ± 18.23 seconds, whereas the control group cried for 98.43 ± 17.33 seconds, indicating the effectiveness of the intervention in reducing pain while undergoing heel lance.

**Table 3:** Comparison of the mean and standard deviation of the duration of crying in the intervention and control groups during both heel prick sessions Values denote mean ± standard deviation.

Heel prick	Intervention	Control
	Mean±SD	Mean±SD
First heel prick	73.14±26.47	112.85±17.99
Second heel prick	67.42±18.23	98.43±17.33

Table 4 depicts that the Friedman test revealed a statistically significant difference in heart rate across pre-test and post-test phases within both the intervention and control groups during the first and second heel lances (p < 0.01).

**Table 4:** Friedman repeated measures comparison of heart rate within the intervention and control groups during both heel prick sessions Mean rank refers to the average rank assigned to the values of each group across different time points or conditions. A p-value < 0.05 is statistically significant.

Heel prick	Groups	Pre-test	Post-test I	Post-test II	Post-test III	Chi-square	p-value
		Mean rank	Mean rank	Mean rank	Mean rank		
First heel prick	Intervention	1.43	4.00	2.99	1.59	193.82	<0.01
	Control	1.01	3.99	2.96	2.03	206.76	<0.01
Second heel prick	Intervention	1.51	4.00	2.94	1.56	194.85	<0.01
	Control	1.04	3.95	2.98	2.04	202.82	<0.01

Table 5 reveals that the Friedman test showed a statistically significant difference in oxygen saturation across pre-test and post-test phases within the intervention and control groups during the first and second heel pricks (p < 0.01).

**Table 5:** Friedman repeated measures comparison of oxygen saturation within the intervention and control groups during both heel prick sessions Mean rank refers to the average rank assigned to the values of each group across different time points or conditions. A p-value < 0.05 is statistically significant.

Heel prick	Groups	Pre-test	Post-test I	Post-test II	Post-test III	Chi-square	p-value
		Mean rank	Mean rank	Mean rank	Mean rank		
First heel prick	Intervention	3.44	1.01	2.06	3.49	195.27	<0.01
	Control	3.91	1.06	2.08	2.95	197.11	<0.01
Second heel prick	Intervention	3.25	1.03	2.27	3.45	181.64	<0.01
	Control	3.73	1.06	2.23	2.98	175.19	<0.01

Table 6 presents the Friedman repeated measures comparison of respiratory rate between the intervention and control groups during the first and second heel pricks. The results show significant changes in respiratory rate across all post-test time points for both groups, with p-values less than 0.01 for both sessions. The mean ranks indicate that the intervention group generally experienced more stable respiratory rates compared to the control group across the heel prick sessions.

**Table 6:** Friedman repeated measures comparison of respiratory rate within the intervention and control groups during both heel prick sessions Mean rank refers to the average rank assigned to the values of each group across different time points or conditions. A p-value < 0.05 is statistically significant.

Heel prick	Groups	Pre-test	Post-test I	Post-test II	Post-test III	Chi-square	p-value
		Mean rank	Mean rank	Mean rank	Mean rank		
First heel prick	Intervention	1.71	3.91	2.81	1.58	172.89	<0.01
	Control	1.14	3.76	3.01	2.09	183.84	<0.01
Second heel prick	Intervention	2.08	3.94	2.49	1.49	166.39	<0.01
	Control	1.26	3.77	3.01	1.95	180.91	<0.01

Table 7 presents the Wilcoxon pairwise comparison of vital parameters for the first and second heel pricks, revealing significant changes in HR, RR, and SaO₂ within both the intervention and control groups. For both heel pricks, the intervention group exhibited larger and more consistent reductions in HR, RR, and SaO₂ with Z-scores ranging from -7.00 to -7.66 and p-values less than 0.01, reflecting significant improvements. The control group also showed significant changes, but with slightly smaller effect sizes (Z-scores between -5.54 and 7.35). These findings suggest that the intervention group experienced more pronounced improvements in vital parameters compared to the control group, with all changes being statistically significant across both time points.

**Table 7:** Wilcoxon signed-rank test comparing the pre-test and post-test differences in vital parameters across multiple time points among neonates in the intervention and control groups Z-value refers to a standardized test statistic used to determine the significance of differences between paired observations. A p-value < 0.05 is statistically significant. r-value: effect size; VP: vital parameters; HR: heart rate; RR: respiratory rate; SaO_2_: oxygen saturation

Heel prick	VP	Pre-test vs. post-test I						Pre-test vs. post-test II						Pre-test vs. post-test III					
		Intervention			Control			Intervention			Control			Intervention			Control		
		Z-score	p-value	r-value	Z-score	p-value	r-value	Z-score	p-value	r-value	Z-score	p-value	r-value	Z-score	p-value	r-value	Z-score	p-value	r-value
First	HR	-7.29	<0.02	-0.87	-7.27	<0.01	-0.86	-7.27	<0.01	-0.86	-7.26	<0.01	-0.86	-7.84	<0.01	-0.93	-7.18	<0.01	-0.85
	RR	-7.26	<0.01	-0.86	-7.30	<0.01	-0.86	-6.15	<0.01	-0.86	-7.11	<0.01	-0.85	-7.01	<0.01	-0.83	-6.67	<0.01	-0.79
	SaO_2_	-7.35	<0.01	-0.87	-7.31	<0.01	-0.86	-7.66	<0.01	-0.87	-7.17	<0.01	-0.85	-6.90	<0.01	-0.82	-6.89	<0.01	-0.82
Second	HR	-7.27	<0.01	-0.86	-7.26	<0.01	-0.88	-7.18	<0.01	-0.85	-7.27	<0.01	-0.86	-7.27	<0.01	-0.86	-7.00	<0.01	-0.83
	RR	-7.41	<0.01	-0.88	-7.41	<0.01	-0.88	-7.57	<0.01	-0.90	-7.01	<0.01	-0.83	-5.77	<0.01	-0.69	-5.67	<0.01	-0.67
	SaO_2_	-7.27	<0.01	-0.86	-7.35	<0.01	-0.87	-6.00	<0.01	-0.71	-6.18	<0.01	-0.72	-5.90	<0.01	-0.70	-5.54	<0.01	-0.66

## Discussion

Heel prick is a routine procedure in neonates during their hospitalization in NICUs, often resulting in pain and discomfort. Pharmacological measures, such as opioids, are commonly administered for pain management in intensive care settings. However, there is a lack of widespread and effective pain relief interventions for less critically ill neonates. Consequently, identifying a simple and acceptable method for enhancing comfort is imperative for both medical and ethical reasons. An ideal analgesic method or drug for neonates should be user-friendly, well-tolerated, minimally invasive, have a rapid onset of action, and exhibit minimal adverse effects. The study shows that 25% dextrose is more effective in stabilizing physiological parameters caused by heel pricks.

The findings of the present study showed no significant differences between the intervention and control groups for most of the variables, including day of life, gender, birth weight, head circumference, length, and birth order (p > 0.05). However, significant differences were observed in APGAR scores at zero and five minutes (p < 0.01) and reasons for admission (p = 0.05), which highlighted variations in neonatal characteristics and conditions between the groups. Similar findings have been reported in multiple studies conducted in India [19-23], which showed that neonatal characteristics like gestation, birth weight, mode of delivery, and gender distribution were comparable across the groups with no statistically significant differences (p > 0.05), and APGAR at five minutes showed no comparison between the groups (p < 0.05).

Vital parameters like heart rate, respiratory rate, and oxygen saturation are key indicators of neonatal physiological stability. They help assess intervention effectiveness, detect early distress, and ensure well-being. During painful procedures like heel pricks, fluctuations in these parameters provide valuable insights into neonatal pain and comfort. The findings of this study are consistent with research in Telangana, India, which compared expressed breast milk and 10% dextrose during heel prick. Their study found that pre-procedure heart rate was significantly higher in the expressed breast milk group (p = 0.0482), while heart rate at four minutes post-procedure was significantly lower in the dextrose group (p = 0.02043) [24]. However, this study contrasts with the study conducted in Imphal, which found no significant difference in post-procedure heart rate between expressed breast milk and oral glucose (p = 0.110) [25]. Similarly, a study conducted in Turkey reported no significant differences in baseline vital signs between groups, with both groups showing increased heart rate and decreased oxygen saturation post-procedure (p > 0.05) [26]. In the present study, although respiratory rate was measured as a vital parameter before and after the heel prick procedure, no prior studies were found that specifically compared changes in respiratory rate following the administration of 25% dextrose or expressed breast milk during heel prick in neonates. Most existing literature has primarily focused on evaluating heart rate, oxygen saturation, and behavioral pain responses, such as crying time or pain scores. The lack of published data on respiratory rate limits direct comparison of our findings, indicating a potential gap in neonatal pain research and highlighting the novelty of including respiratory rate as an objective physiological indicator in this context.

The duration of crying is another crucial indicator of neonatal pain and discomfort, as prolonged crying reflects heightened distress. Along with vital parameters like heart rate, respiratory rate, and oxygen saturation, it plays a significant role in evaluating the effectiveness of pain management interventions. Observing changes in crying duration helps to understand the neonate's response to painful stimuli, assess their comfort level, and make informed decisions about improving neonatal care. The findings of the present study showed a shorter duration of crying in the intervention group, which is similar to the findings of the study conducted in India, which showed a longer crying duration in the expressed breast milk group (4.20±3.31 minutes) compared to 25% dextrose (5.32±0.11 minutes) [27]. In addition, similar findings can be seen in another study conducted in India, which showed 74.80±10.96 seconds of crying duration in the dextrose group compared to the expressed breast milk group, i.e., 104.56±9.16 seconds [28].

Limitations

The study focused on the term neonates and excluded the preterm neonates <37 weeks of gestation; therefore, the effectiveness in those preterm neonates who undergo more painful and repeated procedures is not addressed. In addition, the limited sample size might reduce the generalizability of the findings to a broader neonatal population. Future studies with larger, more diverse samples are recommended to validate and strengthen the results observed in the present study.

## Conclusions

Physiological indicators such as heart rate, respiratory rate, oxygen saturation, and crying duration serve as important measures of neonatal stability and pain response. This study underscores the relevance of these parameters in assessing the effectiveness of non-pharmacological interventions during minor procedures like heel pricks. The results indicate that both 25% dextrose and expressed breast milk contribute to stabilizing these physiological measures, as reflected by reduced crying time. However, 25% dextrose was found to promote quicker stabilization compared to expressed breast milk. Integrating these safe and cost-effective interventions into neonatal care routines can enhance pain management strategies, ultimately supporting greater comfort and well-being in newborns.
